# DAYS AND NIGHTS OF DYADS: EXAMINING BEHAVIORAL INTERDEPENDENCE IN CO-RESIDING COUPLES USING ACTIGRAPHY

**DOI:** 10.1093/geroni/igz038.2818

**Published:** 2019-11-08

**Authors:** Lyndsey M Miller, Zachary Beattie, Nora Mattek, Nicole Sharma, Katherine Wild, Jeffrey Kaye

**Affiliations:** Oregon Health & Science University, Portland, Oregon, United States

## Abstract

Physical activity and sleep are behaviors that contribute to overall health, and are notoriously challenging to improve. For individuals co-residing as a couple, there is growing recognition of the importance of the interdependence between individuals’ behaviors (e.g. the physical activity and sleep habits of one’s partner has an influence on one’s own sleep and physical activity). Yet, studies examining this phenomenon have primarily been conducted among young adults and have either used self-report or episodic measures. The purpose of this study was to examine physical activity and sleep as interdependent behaviors within older adult couples. Using intensive longitudinal data (continuously collected over 35 days) from actigraph watches worn by 54 dyads (mean age in years 72.4 ± 7.1), we examined daily step counts and nightly sleep duration (total of 3780 observations) as a dyadic daily process. Results from multilevel process models for distinguishable dyads (Mplus version 8.2: Muthén & Muthén, 1998-2018) indicated that step count and sleep duration were interdependent processes at the within-dyad level (ps < 0.001). At the between-dyad level, on days with a typical number of steps, on average dyad members’ sleep duration was 7.55 hours [95% CI 4.27 - 10.79 hours], and across dyads, partners’ sleep duration and step counts were also interdependent processes (ps < 0.05). In order to improve the physical activity and sleep of older adults who co-reside with a partner, these results suggest that targeting the dyad, rather than the individual, may be necessary.

